# Uterine Rupture after Laparoscopic Myomectomy in Two Cases: Real Complication or Malpractice?

**DOI:** 10.1155/2017/1404815

**Published:** 2017-12-20

**Authors:** Antonella Vimercati, Vittoria Del Vecchio, Annarosa Chincoli, Antonio Malvasi, Ettore Cicinelli

**Affiliations:** ^1^Department of Biomedical and Human Oncological Science (DIMO), 2nd Unit of Obstetrics and Gynaecology, University of Bari, Bari, Italy; ^2^Santa Maria Hospital, GVM Care & Research, Bari, Italy

## Abstract

We describe two cases of uterine rupture in pregnancy after laparoscopic myomectomy and analyze all the aetiological factors involved in this circumstance according to the recent literature, focusing above all on the surgical procedures and the characteristics of the excised myomas. The two cases of uterine rupture in pregnancy following laparoscopic myomectomy occurred at 36 and 18 weeks of gestation, respectively. Both women had undergone laparoscopic multiple myomectomy and uterine rupture occurred along the isthmic myomectomy scars, despite the fact that compliance with all the recent technical surgical recommendations for the previous laparoscopic multiple myomectomy had been fully observed. In our cases we identified the isthmic localization, size of the excised myomas (≥4 cm), and individual characteristics of the healing process as possible risk factors for “a real complication.” Larger studies and robust case-control analyses are needed to draw reliable conclusions; special care should be paid when performing laparoscopic myomectomy in women planning a later pregnancy.

## 1. Introduction

Uterine rupture in pregnancy is a rare and often catastrophic complication with a high incidence of fetal and maternal morbidity and mortality. The rate of uterine rupture is known to increase in patients with a history of uterine surgery, such as cesarean section and abdominal or laparoscopic myomectomy, but it can also occur in women with a native, unscarred uterus. Laparoscopic adenomyomectomies are widely performed to treat or palliate symptoms such as abnormal uterine bleeding, dysmenorrhea, pelvic and lower abdominal pain or discomfort, urinary bladder irritability, bowel dysfunction, subfertility, pregnancy complications, and pregnancy loss [1, 2]. The natural history of pregnancies following laparoscopic myomectomy is not well understood. It has been hypothesized that uterine rupture following laparoscopic myomectomy is the result of suboptimal healing, coupled with the relatively poor vascularisation of some parts of the uterus, predisposing those sites to weak scar formation after certain types of electrosurgery [3]. In comparison with abdominal myomectomy, the laparoscopic procedure is associated with less postoperative pain, a short hospital stay, and faster recovery time [4, 5]. The frequently reported complications of laparoscopic surgery generally arise due to failure to adequately suture myometrial defects, poor hemostasis with subsequent hematoma formation or excessive use of monopolar or bipolar electrosurgery, and hence devascularization of the myometrium, which can interfere with myometrial wound healing and increase the risk of rupture [6]. Uterine rupture refers to a complete separation of all the uterine layers [7] and of the overlying visceral peritoneum and is often associated with clinically significant paroxysmal pain, uterine bleeding, fetal distress, and even protrusion or expulsion of the fetus and/or placenta into the abdominal cavity. It entails the need for prompt cesarean delivery, uterine repair, or hysterectomy. From the time of diagnosis to delivery, generally only 10–37 minutes elapse before clinically significant fetal morbidity becomes inevitable. Fetal morbidity occurs as a result of catastrophic hemorrhage, fetal anoxia, or both. The diagnosis of uterine rupture is made by clinical observation and can be confirmed by ultrasound imaging. Several aetiological factors have been identified, and here we report our experience of two cases of uterine rupture after previous laparoscopic myomectomy, focusing on the characteristics of the surgery, and the type, localization, and size of the fibroids. In this regard, the new US classification of myomas, MUSA 2015 (morphological uterus sonographic assessment; [8, 9]) introduced to define and standardize US imaging of uterus fibroids, could be useful to better correlate the localization and characteristics of myoma before laparoscopy and reduce the risk of rupture in pregnancy. We considered the site of fibroids according to this classification as G0 = pedunculated intracavitary; G1 = submucosal < 50% intramural; G2 = submucosal ≥ 50% intramural; G3 = 100% intramural, but in contact with the endometrium; G4 = intramural; G5 = subserosal ≥ 50% intramural; G6 = subserosal < 50% intramural; G7 = subserosal pedunculated; G8 = other (e.g., cervical, parasitic).

## 2. Cases Presentation

**Figure 1:**
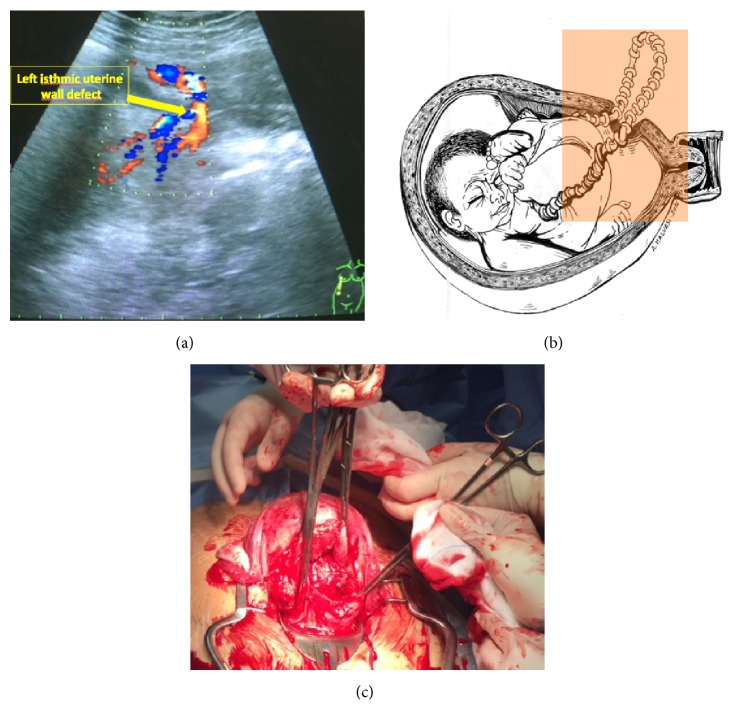
(a) 2D transabdominal US and color Doppler findings: a loop of umbilical cord was noted outside the uterus and running through the left isthmic uterine wall focal defect. (b) Graphic depiction of the loop of umbilical cord herniated outside the uterus through the left isthmic focal defect. (c) Macroscopic appearance of the uterine rupture at surgery.

In the last five years of our clinical and surgical activity 3800 cesarean deliveries (CS) have been performed and in 57 cases (1.5%) cesarean deliveries followed a laparoscopic myomectomy. We report the only two cases (3.5%) of uterine rupture that occurred among these 57 CS following laparoscopic myomectomy. The first case was a 31-year-old woman para 0/0/1/0 hospitalized for abdominal pain of sudden onset in the 34th week of gestation [10]. She had undergone laparoscopic multiple myomectomy 2 years earlier, when different types of myoma were excised: one intramural- (IM-) G4 myoma of the posterior wall of the uterus with a mean diameter (diam.) of 5 cm, one IM-G5 left isthmic myoma (diam. 3 cm), one IM-G4 on the fundus (diam. 3 cm), one subserosal- (SS-) G6 of the right wall (diam. 2 cm), and two IM-G4 of the anterior wall of the uterus (diam. 2 cm each one). At the first clinical examination, the findings were deep abdominal pain, dysuria, and a positive Giordano's sign on the right. Her blood pressure was 132/66 mmHg and heart rate 77 beats/min. She was afebrile and not pale. The fetus was alive at cardiotocographic evaluation. There were no palpable uterine contractions, although the patient was groaning with pain. The cervix was closed and there was no evidence of vaginal bleeding. We performed transabdominal 2D ultrasound that showed an alive intrauterine podalic fetus with normal Doppler flowmetry, oligohydramnios, and minimal intraperitoneal fluid; the patient complained of increasing pain on the left side of the abdomen, where ultrasound revealed the presence of a vascularised area whose venous and arterial flow seemed to be in continuity with the umbilical cord and had the same ultrasound characteristics (Figure 1(a)). This vascularised area was located outside the left wall of the uterus and was likely an early sign of uterine rupture (Figure 1(b)). The breach seemed to be 2 cm long on the left wall of the uterus, at the level of one of the previous laparoscopic myomectomy wounds. Continuous cardiotocographic assessment showed a normal fetal heartbeat and the absence of uterine contractions, but the patient continued to complain of abdominal discomfort and started vomiting. An emergency laparotomy was performed. Surgical findings included a breach running horizontally through the entire anterior wall of the uterus (Figure 1(c)), a moderate quantity of hemoperitoneum, while the fetus had turned so that the left shoulder was facing the abdominal cavity. The baby was delivered alive with the placenta, and no emergency procedure was required. The tear was repaired and the hemoperitoneum drained. The patient made satisfactory clinical progress and was discharged home with a healthy baby on the fifth postoperative day; neonatal follow-up was regular.

**Figure 2:**
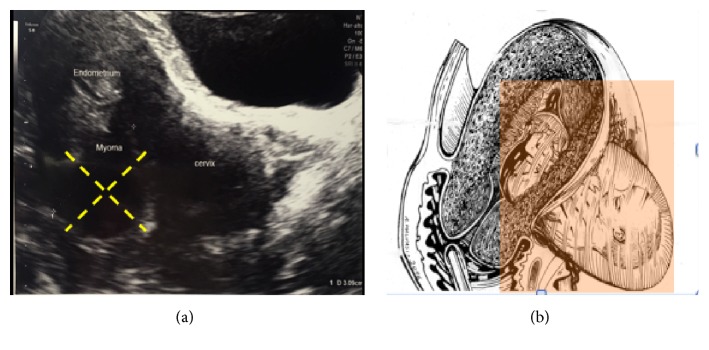
(a) 2D transvaginal ultrasound of the IM isthmic myoma type 5. (b) Graphic depiction of the herniated fetus through the isthmic defect on the previous myomectomy scar.

The second case was a 37-year-old woman, para 0/0/0/0, at 18 weeks of gestation, referred for pregnancy termination due to fetal abnormalities. She, too, had undergone laparoscopic multiple myomectomy 3 years earlier, when two types of myomas were excised: an IM-G5 left isthmic myoma (4 cm) (Figure 2(a)) and a SS-G6 myoma on the fundus (5 cm).

Abortion was induced with vaginal prostaglandin suppositories. Three hours after the administration of the third suppository, the patient began to complain of deep, persistent abdominal pain. On examination, the abdomen was tender, the cervix 2 cm was dilated, and there was evidence of vaginal bleeding. Her blood pressure was 80/50 mmHg and heart rate 120 beats/min. Because of the increasing pain, not correlated with fetal expulsion, and of the worsening clinical conditions, we performed transabdominal 2D ultrasound that showed, inside the peritoneal cavity, herniation of the amniotic sac and fetus through the uterine wall along the previous isthmic laparoscopic myomectomy scar (Figure 2(b)).

The treatment team decided to proceed with laparotomy under general anesthesia. The entire amniotic sac containing the fetus protruded through uterine isthmic breach and about 800 mL hemoperitoneum was detected and drained. The amniotic fluid was clear and odor-free; the placenta was located on the fundus and removed. The tear was repaired in two layers and the patient was discharged 4 days later.

In both cases, the previous laparoscopic myomectomy recording was examined together with the surgeon. There had been no mistakes in the surgical technique related to uterine closure: multiple layer suturing (three-layer) had been performed, the electrosurgery (bipolar coagulation) was gentle, there was no excessive bleeding, and entry into the endometrial cavity had been avoided. Moreover, the two patients had suffered no postoperative complications such as hematoma or infections, which could have interfered with correct wound healing. Compliance with all the recent technical surgical recommendations had been observed.

In the remaining 55 cases of CS after a previous myomectomy, who had suffered no complications in the following pregnancy, the previous myoma was single in 20 cases with the following characteristics: 4 G2 (3 with a mean diameter > 4 cm; no case with an isthmic site); 6 G3 (2 with a mean diameter > 4 cm, 1 isthmic myoma with a diameter of 2 cm); 5 G4 (4 with a mean diameter > 4 cm and no case of isthmic myoma); and 5 G5 (all with a diameter > 5 cm and no case of isthmic myoma). In the other 35 cases of different-sized multiple myomas in various sites, only in one case was the myoma, with a mean diameter of 1.5 cm, localized in the isthmic area. No statistically significant conclusions can be drawn due to the small sample size.

## 3. Discussion

Rupture of a pregnant uterus is one of the life-threatening complications encountered in obstetric practice. Although it may occur in an unscarred uterus, the most common cause of uterine rupture is splitting of a previous cesarean scar. Several aetiological factors may be responsible for rupture of the uterus, including previous cesarean section or laparotomic/laparoscopic adenomyomectomy, trauma, uterine overdistension, uterine anomalies, placenta percreta, and choriocarcinoma [11, 12]. Uterine rupture implies a defect in the uterine musculature, with extravasation of fetal parts and intra-amniotic contents into the peritoneal cavity [13, 14]. It is difficult to make a realistic estimation of the rate of uterine rupture after laparoscopic myomectomy. Several recent studies including large numbers of cases have reported on the efficacy of laparoscopic myomectomy: in a multicenter study, Sizzi et al. evaluated 2050 operations and reported 1 rupture among 386 pregnancies; Malzoni et al. evaluated 982 operations and reported no uterine ruptures [1]. In our experience there seems to be a higher prevalence of uterine rupture after previous laparoscopic myomectomy (3%) but it must be remembered that one of the two cases occurred during the induction of pregnancy termination, in a high risk condition. Our series is still too small for a realistic estimation of the risk.

Uterine rupture after laparoscopic myomectomy is one of the complications of the procedure. It depends on wound healing that can be affected by various factors such as the method and tools used for uterine incision, unsuccessful hemostasis and closing of the myometrial defect [15], the extent of local tissue destruction, the potential formation of infection or hematoma within the myometrium, gas pneumoperitoneum in laparoscopic procedures, and, finally, the individual characteristics of the healing process related to the production of growth factors or excess collagen deposition [6]. Meticulous closure of the myometrial bed following myomectomy can be difficult via laparoscopy and could therefore interfere with the integrity of the scar [3]. Uterine rupture during pregnancy seems to occur more frequently as a consequence of laparoscopic than laparotomic myomectomy [2–16], although this finding is extremely limited because it depends on few reported cases (such as our submitted cases) and has provoked debate in the recent literature. After abdominal myomectomies the scars are of similar thickness to normal myometrium, whereas after laparoscopic procedures they are strained, more contracted, and thinner than normal myometrium. These differences are likely due to the use of sutures to achieve hemostasis during abdominal myomectomy, versus bipolar coagulation during laparoscopic myomectomy. In the latter, the resulting thermal damage to the myometrium induces a proliferation of connective tissue, which cannot undergo remodeling during pregnancy [6]. Malvasi et al. evaluated the presence of neuropeptide substance P and vasoactive intestinal peptide in the pseudocapsule of uterine myomas. Because these neuropeptides may affect wound healing and myometrial function in a subsequent pregnancy, the pseudocapsule neurovascular bundle should be carefully treated, avoiding damaging practices such as an extensive use of coagulation [1]. A study of uterine wound healing using magnetic resonance imaging demonstrated completion of the uterine healing process at 12 weeks after abdominal myomectomy in the absence of hematoma or oedema formation in the myometrium [6]. A study of laparoscopic myomectomy scars identified hematomas in 74% of women one day after surgery, probably due to closure of the uterine defect with only a single layer of sutures. Expert opinion recommends that intraoperative strategies to reduce uterine rupture in subsequent pregnancies include multilayer uterine closure, avoidance of entry into the endometrial cavity, avoidance of excessive electrosurgery to reduce devascularization, and the prevention of haematoma formation, which may affect wound strength [17, 18]. In our experience of two cases there were no surgical laparoscopic factors related to the risk of uterine rupture.

The size and number of the myomas removed, whether entering the endometrial cavity or not, have also been identified as potential factors affecting the risk of uterine rupture in subsequent pregnancies. In the seven uterine ruptures described by Pistofidis et al., the maximum myoma diameter was 4.4 cm, while in 5 patients it was ≤5 cm. Six patients had a single myoma. Only one patient had an intramural myoma, while all the others had subserosal and/or pedunculated myomas. In the research carried out by Parker et al. who described nineteen cases of uterine rupture, the greatest diameter of dissected myomas ranged from 1 to 11 cm (mean, 4.5 cm) [6]. Two small myomas (both 1.2 cm) were removed in 1 woman and 1 myoma in all the others. Pedunculated subserous myomas were removed in 4 women, subserosal myomas in 5, and intramural myomas in 10 [6]. In both our reported cases, the size (>4 cm), isthmic localization, and level of involvement of the myometrial wall (>50%, IM myoma-G5) [9] seem to be risk factors for uterine rupture. The MUSA classification [8] can be considered to better classify myomas before adenomyomectomy and to better predict the risk of uterine rupture in subsequent pregnancies, regarding site and size of myoma. However, due to limited evidence in this field, these factors remain a topic for debate [17].

In conclusion, despite evidence that laparoscopic myomectomy is associated with remarkable benefits, there is one concern that has still to be resolved: is this procedure associated with a higher risk of subsequent uterine rupture compared with open (via laparotomy) myomectomy? Excessive use of diathermy for hemostasis should be avoided and multiple layer suturing should always be done to repair the myometrial defect in cases of deep intramural and subserosal myomas. Our cases of uterine rupture despite observance of all the recent technical surgical recommendations during the previous laparoscopic multiple myomectomy underline the point. Larger studies are needed to better understand whether uterine rupture after laparoscopic myomectomy is a “real” complication of the procedure or a possible “malpractice.”
